# Exploring the health of child protection workers: A call to action

**DOI:** 10.34172/hpp.2022.50

**Published:** 2022-12-31

**Authors:** Javier F. Boyas, Debra Moore, Maritza Y. Duran, Jacqueline Fuentes, Jana Woodiwiss, Leah McCoy, Antonella Cirino

**Affiliations:** ^1^Troy University, School of Social Work and Human Services, 112A Wright Hall, Troy, AL, 36082, USA; ^2^University of Georgia, School of Social Work, 279 Williams St., Athens, GA, 30602, USA

**Keywords:** Secondary trauma, Child protective services, Health behaviors

## Abstract

**Background:** This exploratory study determined if a relationship exists between secondary traumatic stress (STS) related to health status, health outcomes, and health practices among child protection workers in a Southern state.

**Methods:** This study used a cross-sectional survey research design that included a non-probability sample of child protection workers (N=196). Data were collected face-to-face and online between April 2018 and November 2019 from multiple county agencies. A self-administered questionnaire was completed focused on various health behaviors, outcomes, and workplace perceptions.

**Results:** Results of the zero-order correlations suggest that higher levels of STS were significantly associated with not having visited a doctor for a routine checkup (*r*=-0.17, *P*=0.04), more trips to see a doctor (*r*=0.16, *P*=0.01), and increased number of visits to emergency room (ER) (*r*=0.20, *P*=0.01). Lower levels of STS were associated with better self-rated health (SRH) (*r*=-0.32, *P*≤0.001), higher perceptions of health promotion at work (*r*=-0.29, *P*≤0.001), frequent exercise (*r*=-0.21, *P*=0.01), and by avoiding salt (*r*=-0.20, *P*≤0.031). T-test results suggest that workers who did not have children (µ=45.85, SD=14.02, *P*=0.01) and non-Hispanic white workers (µ=51.79, SD=11.62, *P*≤0.001) reported significantly higher STS levels than workers who had children (µ=39.73, SD=14.58) and self-identified as Black (µ=39.01, SD=14.38).

**Conclusion:** Findings show that increased interpersonal trauma was linked to unhealthy eating, general physical health problems, and health care utilization. If not addressed, both STS and poor health and health outcomes can have unfavorable employee outcomes, such as poor service delivery.

## Introduction

 Child protection is one of the more challenging and taxing human service occupations. The nature and organization of the work make child protection inherently strenuous, such as high work demands, low salaries, excessive caseloads, risky and unpredictable case situations, changing policies and standards, on-call duties, understaffed work environments, persistent emergencies, and arduous work schedules.^1-4^ Child protection workers often face the apprehension of making abrupt decisions on complicated cases, sometimes with little to no background information. Such fast-paced decision-making does not always end in selecting the safest option, which has resulted in continuous public and media scrutiny.^5^ It is clear, though, that child protection workers’ decisions are vital, given they are the first line of defense when there is suspicion of child abuse or neglect and effective interventions when cases of abuse or neglect are indicated. Additionally, listening to children talk about traumatic experiences while trying to work in a demanding, challenging, and commonly “insensitive” child welfare structure can potentially put a child protection worker at heightened risk of developing emotional and psychological problems.^6-10^ Thus, for child protection workers to provide quality services to vulnerable populations, they must be mentally, physically, and emotionally prepared.

 Given the magnitude of what is at stake and the number of job-related stressors, it is not surprising that many child protection workers suffer from mental health problems. Such suffering has often resulted in the genesis of adverse occupational stress reactions, such as job stress, burnout, and vicarious trauma.^6-10^ One of the concerning occupational hazards faced by child protection work relates to the increased susceptibility to experiencing vicarious trauma, often recognized as secondary traumatic stress (STS). It has been estimated that as many as 70% of social workers experience STS.^11^ That is because of the multiple times child protection workers are exposed to indirect trauma through a client’s narrative and distressing description of a traumatic event, such as hearing accounts of acts of cruelty, medical neglect, emotional, physical, sexual, and psychological abuse.^12,13^ They also hear and read disturbing content discussed in case reviews and case recordings.^14^Since so many of their clients are survivors of trauma, child protection workers provide empathy for their clients by providing ongoing listening, supporting, and providing various levels of continuing care, which can leave them vulnerable to absorbing the anguish their clients experience.^15^ Not surprisingly, child protection workers too are likely to show stress symptoms of primary trauma.^16,17^ STS is thus viewed as a psychological reaction to a stressor encountered in the workplace associated with the many traumatic accounts shared by trauma survivors.^18^It is well established that STS can manifest on three levels: physical, behavioral, and psychological/emotional.^19^

 Although researchers have documented the psychosocial stress reactions associated with child protection, such as STS, very few studies have explored whether child protection workers’ levels of STS are associated with health status, health practices, and health outcomes. Existing research highlights the relationship between interpersonal trauma exposure and adverse physical health outcomes.^20,21^Furthermore, several studies sampling vulnerable occupational groups revealed that occupational stress can manifest and result in psychosomatic symptoms.^22-26^

 This relationship has been explained by asserting that trauma activates the hypothalamic-pituitary-adrenal axis and the sympathetic nervous systems, which prompts an overreaction in the immune system.^27,28^ This physiological response is attributed to increased ill health and a debilitated immune system.^29^

 We maintain that investigating the global health of this occupational group is highly relevant because, historically, child protection workers hardly ever benefit from organizational health and well-being training. Very few organizations have invested in promoting health and well-being among their workforce. While some child protection organizations have started to introduce programming to address mental health concerns, the same cannot be said about physical health, despite the growing number of studies that underscore the health concerns raised by child protection workers.^30-32^ Current research suggests that child protection workers develop unhealthy behaviors due to the stress and demands of child protection practice, which include unhealthy eating, substance abuse, self-neglect, and lack of exercise.^30,33^ Thus, child protection workers’ health behaviors and health status should be investigated, given the work conditions they experience. Health is not only a key indicator of social wellness, but it is also essential to the work performance of child protection workers.^34^ Ignoring child protection workers’ health could put the entire child welfare system at risk.^15,35^There is very little information in the literature related to how much health is discussed within child welfare organizations or discussion of the nutritional eating habits of child protection workers. Additionally, although many studies suggest that many child protection workers suffer from STS,^36,37^ few studies have explored whether a correlation exists with health status, health practices, and health outcomes among this vulnerable occupational group. This study’s first aim was to identify child protection workers’ health practices and health status in a Southern state. The second aim was to determine if a significant correlation exists between STS, health behaviors, and health outcomes.

## Materials and Methods

###  Research design and sampling 

 This study used a cross-sectional, retrospective research design. A non-probability sample of child protection workers was recruited to participate in this study using a combination of convenience and snowball sampling techniques. The study eligibility criteria were: (1) be at least 18 years of age, (2) be employed in a child protection public agency in a specific Southern state and (3) have no cognitive limitations. Participants were excluded if they worked for a private child protection agency or were public child protection in other neighboring states. No potential participant was left out due to exclusion criteria.

###  Procedures

 Participants were recruited via Facebook, word of mouth, and at trainings targeting child protection workers. Data were collected face-to-face and online from April 2018 through October 2019 from various county agencies across one state. A self-administered questionnaire took approximately 30 minutes to complete. In all, 40 persons opted to complete the survey online, whereas the other 158 participants completed their surveys face-to-face. No significant differences in health and health outcomes were identified between respondents who completed the survey online compared to those who completed the survey in person. Pilot testing with 15 possible participants was conducted to further maximize the face validity of the questionnaire. Their responses were not included in the current analysis. As a result of feedback gained from the pilot testing, the final instrument was abbreviated to reduce the possibility of the participants experiencing mental fatigue. Written informed consent was obtained from all participants. Participants were not given a research honorarium. The Institutional Review Board at the University of Mississippi approved the protocols (protocol number: 18x-288)used in the present study to ensure minimal risk to participants.

###  Instrumentation

 The STS symptoms scale^36^ is a self-report questionnaire consisting of 17 items. Responses are based on a 5-point Likert-type scale (1 = never to 5 = very often). The STS includes three subscales: intrusion (5 items), avoidance (7 items) and arousal (5 items). Items were summed to create a total score, with higher scores indicating a higher level of STS. A total score of 38 or higher indicates STS.^36^ The STS scale had a high internal consistency in the present study (Cronbach’s alpha = 0.89).

 Self-rated health (SRH) continues to be widely used in health surveys because it is considered a robust global measure of general health status in epidemiological studies and an independent predictor of morbidity and mortality.^38,39^ SRH was a single item that asked respondents to answer the question, “How would you describe your overall state of health these days?” SRH was measured as a five-category ordinal variable: poor = 0 to excellent = 4.

 Several measures were included to determine child welfare workers’ health status, health practices, and health outcomes. Chronic health conditions were an eight-item measurement in which workers were asked to report the chronic conditions they were experiencing. Workers were asked if they had been diagnosed with the following conditions: diabetes, heart disease, obesity, asthma, hypertension, cancer, stroke, and liver disease. Each question was a dichotomous variable, indicating either presence (= 1), or absence (= 0) of the diagnosis in question.

 Exercise activity was a single item measure that asked participants how often they managed to get the recommended amount of exercise: 1 = never to 3 = three times a week. Body mass index was calculated by height and weight of respondents. Smoking was a five-item measure that captured participants’ smoking status, how many cigarettes they smoked daily, weekly, and monthly, and if they began smoking as a result of their job. Smoking status was captured as a dichotomized measure: no = 0, yes = 1.

 Nutritional eating was an eight-item measure that asked participants how often they engaged in healthy eating by eating fruit, eating vegetables, eating healthy options, avoiding salt, eating bran, avoiding fried food, avoiding desserts, and avoiding sugary drinks. Each nutritional eating question was an ordinal variable that was measured as Never/rarely = 1, A few times per week = 2, Once a day/every meal = 3.

 The following individual and demographic data were collected: race/ethnicity, age, education level, job titles, marital status, and children. Race/ethnicity was a single item that asked participants to state with which ethnic group they identify. The original options included Caucasian, African American/Non-Hispanic Black, Hispanic/Latino, Asian American/Asian, Native American/Alaskan Native, Hawaiian or Pacific Islander, or not listed. However, since the sample was mostly homogenous, this variable was dichotomized: 0 = non-Hispanic White, 1 = African American/Black. Age was a continuous measure. Educational degree was a nominal measure: Bachelor = 1, Bachelor’s in Social Work = 2, Master’s in Social Work = 3, Master’s in Psychology = 4, Ph.D. = 5, Psy D. = 6, JD = 7, Other = 8. Licensure was dichotomized: No = 0, Yes = 1. Furthermore, job titles were nominal measures: Manager/ Supervisor = 1, Frontline worker = 2, Staff = 3, Legal staff = 4. Marital status: single = 1, married = 2, separated = 3, divorced = 4, widowed = 5. Having children were captured as a dichotomized measure: No = 0, Yes = 1.

 Team health promotion was measured by the Team Health Climate instrument developed by Sonnentag and Pundt.^40^ Three Likert-scale items were used to assess if workers were asked if the topic of health was included in their work meetings, if it was expected within their workplace that they take care of their health, and if there were any exchanges in ideas about healthy living. Child welfare workers responded using a 5-point scale ranging from 1 (strongly disagree) to 5 (strongly agree). All 3 items were aggregated to create the scale, with higher scores representing better perceptions of team health promotion. Previous research of the team health climate scales showed acceptable levels of internal consistency (Cronbach’s alpha = 0.71).^41^ In the present study, Cronbach’s alpha for this scale was 0.86.

 Health care utilization was a five-item measurement. Workers were first asked if they had one person they thought of as their personal doctor or health care provider (1 = Yes, only one, 2 = More than one, and 3 = No, I do not have a personal doctor or health care provider). They were then asked about how long it had been since they last visited a doctor for a routine checkup; this was a Likert scale measure that ranged from 1 = Within the past year to 6 = Never had a routine checkup. The health care utilization measures were three single items previously used in the National Health Interview Survey.^42^ Each item asked participants to report how many times they had visited a health clinic, doctor, and emergency room (ER) in the past 12 months.

###  Data analysis

 Univariate statistics were used to discern the study sample in terms of sociodemographic characteristics. Bivariate analyses included zero-order correlations to determine if multicollinearity was an issue and identify the strength between STS and independent variables. *T*-tests were also used to identify group mean differences by sex and race/ethnicity. Statistical significance was measured at the 95% confidence interval level (*P ≤*0.05). All statistical analyses were conducted using IBM SPSS version 25 (IBM Corp., Armonk, NY, USA).

## Results

###  Descriptive sample information

 The majority of the child protection workers in the sample were Black/African American (*n* = 152; 77%), followed by non-Hispanic White/Caucasian (*n* = 44; 22%). The sample participants identified primarily as female (*n*= 197, 97.5%). The median age of the respondents was 36.86 (SD = 10.34) years old. In terms of marital status, most of the respondents indicated being single (*n* = 93; 46%), followed by those who were married (*n* = 84; 42%). Many respondents reported having children 73.8% (*n*= 149). In terms of job tenure, respondents reported a mean average of 68.73 months, or 5.72 years of working for the child protection agency. Most respondents were frontline workers (*n* = 136; 67%), whereas the remaining were in managerial and supervisory positions (*n* = 42; 20%). Most respondents held a Bachelor’s in Social Work (BSW) (*n* = 123; 61%), while 50 (25%) reported possessing an master of social work (MSW). Only 33.7% of respondents (*n* = 68) were Licensed Social Workers. Respondents indicated that 63.4% (*n*= 128) worked in a rural community, followed by 22.3% (*n*= 45) of workers who worked in a semi-rural community.

###  Health status and health behaviors 

 In terms of health status, 62% of respondents indicated having either poor or fair health. Respondents reported having been diagnosed with the following chronic health conditions: diabetes 18% (*n* = 37), heart disease 4.5% (*n* = 9), obesity 28.7% (*n* = 19), hypertension 28.7% (*n* = 58), asthma 6.4% (*n* = 13), cancer 1.5 % (*n* = 3), stroke 1% (*n* = 2) and liver disease.5% (*n* = 1). Among the respondents, 29% reported having at least one chronic condition, 13% reported having two conditions, while 8% reported having 3 chronic conditions. Most of the respondents did not smoke cigarettes (*n* = 93%). Seven respondents stated that they started smoking as a result of working at the agency. The mean average body mass index (BMI) of survey respondents was 31.64 (SD = 8.34).

 Participants were asked if they managed to get the recommended amount of exercise per week which is at least 30 minutes three times per week. The majority of respondents (55.4%; *n*= 112) indicated they rarely/occasionally exercised the recommended amount, while 33.7% (*n*= 68), indicated that they never exercised the recommended amount. Only 10.4% (*n*= 21) of the respondents indicated they exercised the sufficient amount/got sufficient physical activity in their work.

###  Nutritional eating 

 In terms of healthy eating, 22.8% (*n*= 46) of the participants indicated that they never/rarely engaged in eating fruits, while 68.4% (*n*= 128) indicated that they engaged in eating fruits a few times per week. Only 12.4% (*n*= 25) indicated that that they engaged in eating fruits with every meal. Regarding engaging in eating vegetables, participants indicated 22% (*n* = 10.9) never/rarely, while 56.9% (*n*= 115) indicated a few times per week, and 29.7% (*n*= 60) indicated eating vegetables once a day/every meal. In terms of avoiding salt, 53.5% (*n*= 108) of participants indicated never/rarely, 31.7% (*n*= 64) indicated a few times per week, 12.4% (*n*= 25) indicated avoiding salt once a day/every meal. Participants were asked how often they ate bran: 57.9% (*n*= 117) indicated never/rarely; 33.7% (*n*= 68) indicated a few times per week; 3% (*n*= 6) indicated once a day/every meal. Participants were also asked how often they avoided fried food, and 39.1% (*n*= 79) reported never/rarely avoiding fried food; 46% (*n*= 93) avoided fried food a few times a week; and 11.9% (*n*= 24) avoided fried food once a day/every meal. Furthermore, 42.6% of participants never/rarely avoided sugary drinks; 39.6% (*n*= 86) avoided sugar drinks a few times per week and 15.8% (*n*= 32) avoided sugary drinks once a day/every meal.

###  Health-seeking behaviors 

 In terms of having a personal care provider, 61% of the respondents reported having one person they considered their personal doctor or health care provider, whereas 14% did not have one person they considered their personal care provider. The majority of respondents, 75%, reported visiting the doctor for a routine doctor visit that did not include an exam for a specific injury, illness, or condition in the past year, while another 13% visited the doctor for a routine visit within the past two years. In terms of health care utilization, respondents were asked how many times they visited a health clinic, a doctor’s office, and the ER. Twenty percent of respondents did not visit the doctor for a routine doctor visit. On average, respondents average 3.39 (SD = 3.85) visits to a doctor’s office, 2.95 (SD = 3.37) visits to a health clinic, and.55 visits to the ER in the past 12 months. Roughly 33% of the respondents reported needing to go to see a doctor but did not do so because of cost.

###  Health promotion in the workplace

 Participants were surveyed about how much health is discussed within their team meetings and other team events. Roughly 46% either strongly disagreed or disagreed that the topic of health is discussed within their team. Moreover, 41% strongly disagreed or disagreed that they exchanged ideas about healthy living within their team. However, 54% strongly agreed or agreed that it is expected that they take care of their health.

###  Bivariate results

 Results of the zero-order correlation suggest that STS is significantly associated with healthy eating by avoiding salt, frequency of exercise, team health climate, frequency of routine check, number of visits to the doctor, number of visits to ER, and SRH. Results of the *t* test suggest that having children and race were significantly associated with STS. In terms of health status, respondents who reported poorer health also reported higher levels of STS (*r* = -0.32, *P* ≤ 0.001). In terms of health behaviors, respondents that did not exercise 30 minutes 3 times weekly reported significantly higher levels of STS (*r* = -0.21, *P* ≤ 0.01). Respondents who reported not avoiding eating salt also reported higher levels of STS (*r* = -0.20, *P* ≤ 0.05). In terms of the workplace, respondents who perceived higher perception of health being promoted amongst their team also reported lower levels of STS (*r* = -0.29, *P* ≤ 0.001). Turning to healthcare utilization, respondents who made more trips to see a doctor (*r* = 0.16, *P* ≤ 0.05) and the ER (*r* = 0.20, *P* = 0.01) also reported higher levels of STS. However, respondents who have not visited a doctor for a routine checkup in a while reported significantly higher levels of STS (*r*= 0.17, *P* = < 0.05). Demographically, *t* test results suggest that workers who had children (µ = 39.73, SD = 14.58) reported significantly lower levels of STS than workers without children (µ = 45.85, SD = 14.02). African American child protection workers (µ = 39.01, SD = 14.38) reported significantly lower levels of STS compared to non-Hispanic White workers (µ = 51.79, SD = 11.62).

## Discussion

 Child protection workers work in highly stressful work environments and are regularly exposed to emotional duress indirectly, which may result in the genesis of interpersonal trauma. The present study sought to explore how STS was associated with multiple health outcomes among child protection workers in a southern state. Among a sample of child protection workers, 57.7% of respondents in the present study reported STS scores of 38 or more. This finding is concerning, given that interpersonal trauma has been linked to somatic symptoms and general physical health problems.^43^If not addressed, STS and poor health can have unfavorable employee outcomes, such as poor service delivery. Thus, if child welfare outcomes are going to improve, improving child protection workers’ health and mental health will be critical.

 Health status was significantly associated with STS. In the present study, results indicate that STS was significantly associated with SRH. In fact, SRH shared the strongest relationship with STS. Consistent with existing studies, poorer health perceptions were significantly associated with increased levels of STS among child protection workers.^21,32,44^ This finding may be related to the correlation between health outcomes and the clustering of avoidance and hyperarousal symptoms that underly STS, resulting in a potentially cumulative adverse impact on health.^32,45,46^ One study suggests that child protection workers’ stress contributes to unhealthy eating habits, disturbed sleep, and substance use and poor health, such as high blood pressure, weight gain, and fatigue.^30^Other studies suggest that secondary stress among social workers was associated with sleep disturbance, sexual difficulties, poor eating habits, and elevated blood pressure.^26^

 Health practices were found to be correlated with STS. The observed relationship between STS and lack of exercise among child protection workers is indicative of literature illustrating the impact of mental health distress on disengaging from physical activity.^30,47^ In one study, child protection workers noted having “no energy” or “being too tired” to engage in physical activity after work, highlighting the emotional exhaustion of STS leading to bodily fatigue.^15^This finding is consistent among first responders who also report lower levels of physical activity when having higher levels of STS.^48^ Moreover, this finding affirms the consequences of trauma on the physical health of child protection workers.

 The present study revealed that respondents who reported not avoiding eating salt also reported higher levels of STS. There currently needs to be more research examining how limiting salt can curtail STS. While it has been noted that psychological dysregulations ensue as a result of normal homeostatic functioning being shifted towards abnormal ranges due to prolonged secretion of stress hormones,^49^ the connections between behaviors associated with these dysregulations as it relates to STS and salt intake have not been established. Furthermore, psychosocial stimulation associated defense responses (stress) have been shown to induce an increase in salt appetite.^50-52^ One suggestion to this occurrence may be that after prolonged salt intake to ameliorate stress activation, individuals may lean on salt to manage stressful situations and become less able to deal with consistent stress, such as work-related stress or STS. More research is needed that elucidates the workings of this relationship.

 Limited literature also exists regarding the association between child protection workers and their participation in routine check-ups and ER visits and doctor visits. Some literature suggests child protection workers miss appointments or come to work sick due to time constraints with their work schedule and fear falling behind on their existing workload.^30,33^ One study suggests child welfare workers directly referenced not feeling they had time to attend routine check-ups due to overwhelming workloads and described preventative care becoming an added stressor.^30^

 Turning to sociodemographics, two were significantly associated with STS. Findings suggest a significant relationship exists between having children and lower STS scores. This may be the case because having loving and supportive relationships with friends and family may increase child protection workers’ capacity to manage different types of stress.^53^ For child protection workers without children, it is reasonable to assume that the absence of psychosocial resources, such as children, does not allow these workers to find the fortification and inner strength to realize mental equilibrium or emotional permanence, which can increase the onset of distress.^54^ Essentially, the missing support from personal resources, such as those with children, can lead to poorer individual coping.^55,56^ However, our results contradict the existing literature, which implies that STS levels are higher among child protection workers who reported having children.^31,57^ One of STS’s notable consequences is strain and withdrawal from personal relationships as a defense mechanism against traumatic experiences shared by clients.^31^ Several participants in James’study^31^ reported experiencing STS had impacted their relationships because of feeling beleaguered by constant distractions and lower moods. Respondents noted their stress levels increased because of not having enough time with family and friends and missing their children’s school events. Still, more research is needed that elucidates possible explanations as to why child protection workers without children suffer from higher levels of STS.

 In this study, race mattered. The racial identity of child protection workers was found to predict increased levels of STS, specifically among White child protection workers. While there is not literature to help explain this finding due to mostly white samples in child welfare studies,^58^ several interpretations can be advanced. This finding could relate to the context of the workers, which is the southern state itself, where data were collected. Historically, and even today, this state’s quest for full integration continues to be a struggle. This is a state with a deep history of enslavement, sharecropping, racial exclusion separation, hate group participation, and aggressive anti-Black activism. This history allowed many Whites to experience life in a cultural vacuum where they were surrounded by white peers.^59^ Understanding this backdrop, it may be that what White child protection workers in this study heard traumatic narratives that they have never heard of or experienced directly. It could be argued that the life experiences by Whites in this Southern state are radically different from the many families of color, and rural populations who receive child protection services. According to constructivist self-development theory, a person constructs their realities based on self-perceptions and schemas, influenced by their lived experiences, that stem from interpersonal, intrapsychic, familial, cultural and social experiences.^19,60^ Black child protection workers in this Southern state may have experienced lower incidences of vicarious trauma because they have been already exposed to accounts of traumatic experiences of child abuse and neglect that fit all too well with their reality. Because of their lived experience, Black child protection workers had already confronted the damage generated by intergenerational trauma, which may have lessened the pain of hearing accounts of trauma among their clients. The traumatic narratives heard by Black child protection workers may have altered their cognitions and worldview in ways that did not materialize for non-Hispanic White child protection workers, who may have never been exposed to the trauma they heard from clients.

 It can also be hypothesized that the deflated account of STS among Black child welfare workers can be attributed to the Black Superwoman Schema (the overwhelming majority of Black respondents were women), which posits that Black women are less likely to report STS due to their socialization, which is linked to racial and gendered schema that includes displaying strength to overcome racial adversity.^61,62^Watson and Hunter^63^ expand on how this multidimensional construct is characterized as an “obligation to manifest strength, emotional inhibition, resistance to utilize mental health self-care resources, rejection of dependence on others, determination to succeed, and caretaking”^63^(p. 445). Qualitative findings suggest Black women, particularly professionals, resist showing vulnerability because they do not want to give their counterparts a sense that they could not do the work, even when working with limited resources,^62^ as is often the case in child protection work.

 Team health climate was also examined in this study, which revealed thatchild protection workers reported lower levels of STS when they perceived working in an environment where team health was being promoted. To the best knowledge of the authors, no other study has examined if a relationship exists between STS and team health climate. One study found that team health climate was generally associated with positive health and mental health.^41^ The results are consistent with broader research suggesting that health climate is a milieu source that enables health-related outcomes among workers.^41,64^ This finding underscores the importance that an organization’s climate can have on serving as a social cue to trigger desired and rewarded health behaviors that safeguard child protection workers from ill health.^64^

###  Implications

 Child welfare workers experience elevated rates, such as 80% mild, 47% moderate, and 22% full STS severity levels.^65^ Due to current reports of STS rates among child protection workers, exploration of how STS impacts the health of child protection workers, as evidenced by the proven association between STS and ER visits, doctor visits, and routine checks, may assist with developing better support systems for this strained workforce that is a crucial base in supporting families and children in need. Child welfare organizations are primed to become distal antecedents for employee health and well-being. To mitigate the genesis of STS, organizations can be intentional about emphasizing concern, care, and consideration about favorable employee health- and mental health-related outcomes. This can be accomplished through using health promotion advocacy to encourage self-care practices among its employees by “maintaining a healthy diet, physical exercise, balancing work and play, rest, spiritual replenishment, and building social networks”.^31^The National Association of Social Workers (NASW)^66^ supports organizational policies that promote self-care, including a healthy diet, adequate sleep, physical activity, health care, and vacations to prevent STS.^67^ This is important given that empirical evidence exists that links self-care practices with more favorable perceptions of health among child protection workers.^68^

 Given the number of poor health habits (e.g., poor nutritional eating habits, low exercise activity levels) reported by child protection workers in this study, child welfare organizations must begin to consider the health status of their workforce. Most of the workers in the present study reported poor or fair health, almost 30% reported suffering from at least one chronic condition, many had very high BMI counts, and over 60% did not have someone they considered their personal doctor or health care provider. Thus, it would be prudent for organizations to take on the role and responsibility of promoting the well-being of their employees.^69^ It has been argued that a positive organizational climate is one that provides learning opportunities and rewards.^64^ Child welfare administrators should consider encouraging teams within the agency to discuss health matters and provide them with health and mental-related information and practical support,^41^ such as trauma-specific workshops or contract or partner with community clinics to host on-site monthly health checkups for the employees. This partnership is germane for child protection staff, given that many respondents stated they could not take time away from work to tend to their medical needs.

 To mitigate the negative impacts of STS, child welfare agency administrative responses should integrate trauma-informed child welfare practices into their organizational culture.^68^ Trauma-informed child welfare practices by staff concede how traumatic events could influence children, families, and child protection professionals who serve them. Still, such an approach should also acknowledge how the workers might suffer from the various traumatic narratives they have encountered while working in child protection.^70^ For example, normalizing the experience of secondary trauma among workers is essential in considering trauma as an underlying explanation for behavioral and emotional distress.^70^ Such a perspective may change the deficit approach child protection workers are met with by their colleagues. The conversation may shift from asking, “What’s wrong with you?” to “What happened to you?”^70^(p. 6).

 Another implication of this study’s findings is the potential that child protection workers’ consumption of salt to manage stress may cause increased tolerance, rendering this coping mechanism less and less effective even with increased intake over time. Once an individual cannot cope with daily life stressors, an allostatic overload can occur.^71^ Allostatic load refers to the cumulative burden of chronic stress and life events.^71^ “Chronic stress and allostatic load shift the operating range of numerous biological systems”^49^(p. 798). These shifts in biology could also play a role in how workers engage with salt intake and utilize salt and nutrition as mitigating factors in coping with chronic stress, job burnout, and STS.^49^ More attention should be given to the allostatic load count of child protection workers due to the connection of chronic stress and allostatic load on the biological makeup of individuals exposed to consistently stressful work environments; the impact of stress on this vulnerable occupational group could dramatically alter their health trajectories in terms of comorbid conditions. This is further evidenced by the vast number of comorbid conditions reported by participants in the current study, such as obesity, hypertension, and diabetes stemming from chronic physical inactivity and heavier salt consumption. Future longitudinal research should be carried out that examines allostatic load counts among child protection workers to determine if the workplace stressors have created significant wear and tear on their bodies since joining the child protection workforce.

 Despite being one of the few studies exploring child protection workers’ health and health outcomes, several limitations should be acknowledged. First, the cross-sectional survey research design does not allow for any causal inferences. Second, it is unknown how many of the workers experienced a personal traumatic event(s) prior to working in these roles. Some research suggests helping professionals have a higher prevalence of individual trauma than other professionals, which may worsen what is experienced in the workplace.^72^ Third, given the topic explored in this study, there is a potential for a self-selection bias. It is possible that the propensity for participating in this investigation was related to a participant’s greater interest in, or direct experience, with personal trauma. Fourth, the sample was somewhat homogeneous because it was predominantly female respondents and primarily African American. Other genders’ and racial/ethnic groups’ perspectives were not captured. Fifth, the generalizability of results are limited due to the convenience sampling methods used within the current study. Last, data were collected from a single southern state, which may not be representative of patterns of STS experienced nationally.

## Conclusion

 The present study underscores the extent to which STS is associated with multiple health behaviors among child protection workers in a southern state. There does appear to be a significant correlation between STS levels, SRH, lack of exercise, salt intake, and healthcare utilization. Moreover, we found that STS levels were lower among child protection workers who believed health promotion was stressed in their team environment. These results underscore the importance of how physical health relates to a worker’s inward dynamics. Thus, more research is needed to identify how public child welfare workers cope with STS to ensure they do not develop unhealthy health habits that can contribute to or expand health disparities among this vulnerable work group. More importantly, public child protection workers who unsuccessfully abate the deleterious consequences of their work on themselves can harm them and place their clients at further injury by not knowing how to respond and redirect their client’s set of circumstances appropriately.^72^ Such oversight has the potential to significantly diminish the service delivery received by the children and families in the child welfare system. As the current study unveils, uncovering the adverse outcomes associated with child protection work becomes critical. It identifies that in our effort as a society to create positive change, we may be establishing a system of oppression under the guise of professionalism and humanitarian effort that disenfranchises a vulnerable work group that is protecting an even more vulnerable population.

## Author Contributions

**Conceptualization:** Javier F. Boyas.

**Data curation: J**avier F. Boyas, Debra Moore, Maritza Y. Duran.

**Formal Analysis: **Javier F. Boyas, Debra Moore, Maritza Y. Duran.

**Investigation: **Javier F. Boyas, Debra Moore.

**Methodology:** Javier F. Boyas, Debra Moore.

**Project administration: **Javier F. Boyas.

**Resources: **Javier F. Boyas, Debra Moore.

**Supervision: **Javier F. Boyas.

**Validation: **Javier F. Boyas.

**Visualization: **Javier F. Boyas, Jacqueline Fuentes, Jana Woodwiss, Leah McCoy.

**Writing – original draft: **Javier F. Boyas, Debra Moore, Maritza Y. Duran, Jacqueline Fuentes, Jana Woodiwiss, Leah McCoy, Antonella Cirino.

**Writing – review & editing: **Javier F. Boyas, Debra Moore, Maritza Y. Duran, Jacqueline Fuentes, Jana Woodiwiss, Leah McCoy, Antonella Cirino.

## Funding

 No external funding was obtained for the current study.

## Ethical Approval

 All procedures performed in studies involving human participants were in accordance with the ethical standards of the institutional and/or national research committee and with the 1964 Helsinki declaration and its later amendments or comparable ethical standards. The procedures carried out in this research were approved by the University of Mississippi’s ethics committee.

## Competing Interest

 The authors declare no conflicts or competing interests.
